# The association between serum uric acid and prognosis of nasopharyngeal carcinoma: a comprehensive meta-analysis of observational studies

**DOI:** 10.3389/fonc.2026.1922633

**Published:** 2026-09-29

**Authors:** Xin Wang, Shukai Zheng, Taifeng Du

**Affiliations:** 1 Medical Record Statistics Office, The First Affiliated Hospital of Shantou University Medical College, Shantou, Guangdong, China; 2 Department of Medical Management, The Second Affiliated Hospital of Shantou University Medical College, Shantou, Guangdong, China

**Keywords:** meta-analysis, nasopharyngeal carcinoma, overall survival, prognosis, serum uric acid

## Abstract

**Background:**

At present, the prognostic value of serum uric acid (SUA) in Nasopharyngeal carcinoma (NPC) remains controversial, and relevant clinical evidence lacks systematic and comprehensive verification. This the first-ever meta-analysis comprehensively synthesized evidence across various regimens to systematically evaluate the association between serum uric acid (SUA) levels and survival outcomes in NPC patients.

**Methods:**

This meta-analysis was strictly performed in accordance with the Preferred Reporting Items for Systematic Reviews and Meta-Analyses (PRISMA) guidelines. Eligible clinical studies focusing on the prognostic role of SUA in NPC were systematically retrieved from domestic and international electronic databases. All retrieved literatures were screened strictly based on predefined inclusion and exclusion criteria. The methodological quality of enrolled studies was assessed using the Newcastle-Ottawa Scale (NOS). Literature screening, data extraction, and quality evaluation were independently conducted by two investigators, and any discrepancies were resolved by consensus or consultation with a third senior researcher. Key prognostic data including hazard ratios (HR) with 95% confidence intervals (CI) for Overall Survival (OS), Progression-Free Survival(PFS), and Distant Metastasis-Free Survival (DMFS) were extracted and pooled for quantitative analysis to evaluate the prognostic significance of SUA in NPC.

**Results:**

The pooled results demonstrated that elevated SUA was significantly correlated with OS in NPC patients, with a combined HR of 0.245 (95% CI: 0.092–0.398). In contrast, no statistically significant associations were observed between SUA levels and PFS or DMFS. The pooled HR for PFS was 0.557 (95% CI: 0.070–1.044), and the pooled HR for DMFS was 0.715 (95% CI: 0.023–1.407). The 95% CI of both PFS and DMFS crossed the invalid value of 1, indicating no significant statistical difference.

**Conclusion:**

SUA level is an independent prognostic factor for OS in patients with NPC, and higher levels of uric acid may improve the overall survival of patients. No significant correlation was identified between SUA levels and PFS or DMFS in NPC patients. SUA may serve as a novel, convenient, and non-invasive biomarker for prognostic evaluation of NPC, may provide a reference for prognostic risk stratification and individualized management. Further large-sample, high-quality prospective cohort studies are warranted to validate the present findings.

**Systematic review registration:**

https://www.crd.york.ac.uk/PROSPERO/view/CRD420261483250, identifier CRD420261483250.

## Highlights

- This is the first comprehensive meta-analysis to systematically and quantitatively evaluate the correlation between serum uric acid (SUA) levels and prognostic outcomes of nasopharyngeal carcinoma (NPC), filling the gap of lacking integrated evidence in this research field.
- Elevated SUA is identified as a significant prognostic indicator for better overall survival in NPC patients, with a robust pooled HR of 0.245 (95%CI: 0.092–0.398), while SUA shows no significant correlation with PFS and DMFS.
- This meta-analysis was strictly performed in accordance with the PRISMA guidelines with rigorous literature screening and quality evaluation. It provides high-quality evidence that SUA may be utilized as a simple, non-invasive auxiliary biomarker for prognostic stratification and clinical management of NPC.

## 1 Introduction

Nasopharyngeal carcinoma (NPC) is a unique epithelial malignancy originating from the nasopharyngeal mucosa, with distinct ethnic and geographical distribution characteristics. The disease exhibits high incidence in southern China, Southeast Asia and other endemic regions, and is characterized by high invasiveness, early distant metastasis and heterogeneous clinical prognosis (1). As documented in the GLOBOCAN 2022 report, nasopharyngeal carcinoma (NPC) remains a non-negligible global public health concern, contributing to 120,416 new confirmed cases and 73,476 disease-specific deaths worldwide in that year (2). Despite the rapid development of comprehensive treatment strategies including radiotherapy, chemotherapy and targeted therapy in recent years, the long-term survival of NPC patients remains unsatisfactory, and individual differences in tumor recurrence and metastasis are prominent. As recommended by current clinical oncology guidelines, induction chemotherapy combined with subsequent concurrent chemoradiotherapy (CCRT) represents the mainstream therapeutic strategy for the management of locally advanced nasopharyngeal carcinoma (3). Epidemiological data further reveals that around 15% of NPC cases are found to have pre-existing metastatic foci in bone, liver and lung at the point of primary diagnosis (4). For the subgroup of metastatic or recurrent NPC patients, 30% of them have a median overall survival of less than 2 years (5). Accurate prognostic evaluation and risk stratification are essential for optimizing clinical treatment regimens and improving survival outcomes of NPC patients. Therefore, exploring effective, convenient and clinically accessible biological markers for prognostic prediction has become a research hotspot in the field of NPC.

Serum uric acid (SUA), the end product of human purine metabolism, is mainly synthesized in the liver, intestine and vascular endothelium, and excreted via the kidneys and intestines (6). Population-based evidence has identified high-purine diets, excessive alcohol consumption, obesity and hypertension as independent risk factors for elevated SUA (7). Such SUA elevation, which can also be triggered by impaired purine metabolism or reduced uric acid excretion, is conventionally associated with hyperuricemia, gout and various metabolic disorders (8, 9). Hyperuricemia is a prevalent chronic condition, defined as a SUA level exceeding 7.0 mg/dL in males and 5.7 mg/dL in females. Epidemiological data from the United States indicates that the incidence of hyperuricemia stands at 20.2% among men and 20.0% among women (10).

In recent years, accumulating studies have confirmed that SUA is closely involved in tumor microenvironment regulation, oxidative stress response, inflammatory reaction and tumor cell proliferation and metastasis. A series of previous studies have consistently reported significant associations between serum SUA concentrations and the incidence of diabetes, cardiovascular disease, kidney disease, and various malignancies (11). Abnormal SUA levels have been proven to have potential prognostic value in a variety of malignant tumors, including colorectal cancer (8), renal cancer (12), prostate cancer (13), colorectal cancer (14). Conventional observational studies have reported that higher serum SUA levels are protective against cancer (15, 16), However, the correlation between SUA level and NPC prognosis remains controversial. Relevant clinical studies have small sample sizes, scattered research conclusions, and lack of unified quantitative evidence, which cannot provide reliable reference for clinical practice.

At present, there is a lack of systematic evaluation of the association between SUA and NPC prognostic outcomes. Therefore, the present study conducted a comprehensive quantitative synthesis of existing published data for the first time. Strictly following standardized meta-analysis specifications, this study systematically evaluated the predictive value of SUA for OS, PFS and DMFS in NPC patients, aiming to provide high-level evidence-based basis for clinical prognostic assessment and individualized management of NPC.

## 2 Methods

### 2.1 Study design

This meta-analysis was conducted in accordance with the PRISMA 2020 statement (17) and was registered in PROSPERO (registration number: CRD420261483250). The PRISMA 2020 checklist is provided in **Supplementary Materials** (PRISMA_2020_checklist). All procedures of literature retrieval, screening, quality evaluation, data extraction and statistical analysis were standardized and predefined to ensure the rigor and reproducibility of the research results.

### 2.2 Literature retrieval strategy

Domestic and foreign mainstream electronic databases were comprehensively searched, including PubMed, Embase, Web of Science and Cochrane Library. The search terms combined subject words and free words, with the key search terms: “serum uric acid”, “SUA”, “nasopharyngeal carcinoma”, “NPC”, “prognosis”, “survival”, “recurrence”, “metastasis”. The retrieval time was from the inception of each database to March 2026, and the language was limited to English. In addition, the references of included literatures were manually traced to avoid missing eligible studies.

### 2.3 Inclusion and exclusion criteria

Inclusion criteria: (1) The study subjects were pathologically diagnosed with nasopharyngeal carcinoma; (2) The study explored the correlation between preoperative or post-treatment SUA level and NPC prognostic indicators; (3) The prognostic outcomes included OS, PFS or DMFS; (4) The study provided available hazard ratio (HR) and 95% confidence interval (CI) for survival analysis; (5) The study type was cohort study or case-control study with complete data.

Exclusion criteria: (1) Reviews, letters, conference abstracts, case reports and animal experiments; (2) Repeatedly published literatures; (3) Studies with incomplete prognostic data or unextractable survival indicators; (4) Patients combined with other malignant tumors, severe metabolic diseases or immune system diseases that affect SUA levels.

### 2.4 Literature screening and data extraction

Two independent researchers (X.W. and S.Z.) completed literature screening and data extraction in strict accordance with the inclusion and exclusion criteria. First, preliminary screening was conducted by reading titles and abstracts, and duplicate and obviously unqualified literatures were eliminated. Then, the full text of the remaining literatures was read for secondary screening. The extracted main data included: basic information of enrolled studies (first author, publication year, region, sample size), clinical baseline characteristics of patients, and core prognostic data including HR and 95%CI of OS, PFS and DMFS related to SUA levels. Any disagreements during the process were resolved by discussion or consultation with a third senior researcher to ensure the accuracy of data extraction.

### 2.5 Quality assessment

The methodological quality of all included observational studies was evaluated using the Newcastle-Ottawa Scale (NOS) (18). The evaluation dimensions included subject selection, group comparability and outcome evaluation, with a total score of 9 points. Studies with a score ≥7 points were regarded as high-quality literature, 4–6 points as medium-quality, and <4 points as low-quality (19). Low-quality studies were excluded from the final meta-analysis to ensure the reliability of pooled results.

### 2.6 Statistical analysis

All statistical analyses in the present meta-analysis were performed using Stata 17.0 software (StataCorp LP, College Station, TX, USA). The pooled hazard ratios (HR) and corresponding 95% confidence intervals (CI) were calculated to quantitatively evaluate the association between SUA levels and prognostic outcomes (OS, PFS, and DMFS) in NPC patients. This study performed a natural logarithm transformation on HR and 95%CI from all included studies to obtain log(HR), and calculated the corresponding standard errors. The effect size distribution was adjusted to conform to the inverse-variance weighted pooling method. After weighted pooling, the results were back-transformed using an exponential function to return to the original HR scale. Statistical inference was conducted using either a fixed-effects or random-effects model, selected based on the Q test and I² statistic. Statistical heterogeneity across the included studies was assessed using the I² statistic and Cochran’s Q test. I² < 50% or P > 0.10 indicated low to moderate heterogeneity, and a fixed-effects model was applied for pooled analysis; otherwise, a random-effects model was adopted (20). A two-tailed P value < 0.05 was considered statistically significant.

Sensitivity analysis was performed by sequentially omitting each individual study one at a time and recalculating the pooled HR and 95%CI. The purpose of sensitivity analysis was to evaluate the stability and reliability of the pooled results and identify whether any single study excessively affected the overall synthetic outcome. Egger’s test was conducted to quantitatively and visually evaluate potential publication bias for each prognostic indicator (21). If the number of included studies is less than 10, no quantitative methods such as Egger’s regression are used to test for publication bias. Instead, potential bias is evaluated by manually checking the characteristics of the literature.

## 3 Results

### 3.1 Selection and identification of studies

Literature screening was strictly conducted in accordance with the predefined retrieval strategy and inclusion and exclusion criteria. A total of 33 relevant articles were initially retrieved from four mainstream electronic databases. After preliminary screening, 19 duplicate articles were excluded. Subsequently, 4 irrelevant articles were eliminated by reading titles and abstracts, 2 articles without available prognostic data were removed, and 4 reviews and conference abstracts were further excluded. Ultimately, a total of 4 eligible studies (22–25) were included in the final meta-analysis. The detailed literature screening process is presented in **Figure 1**.

**Figure 1:**
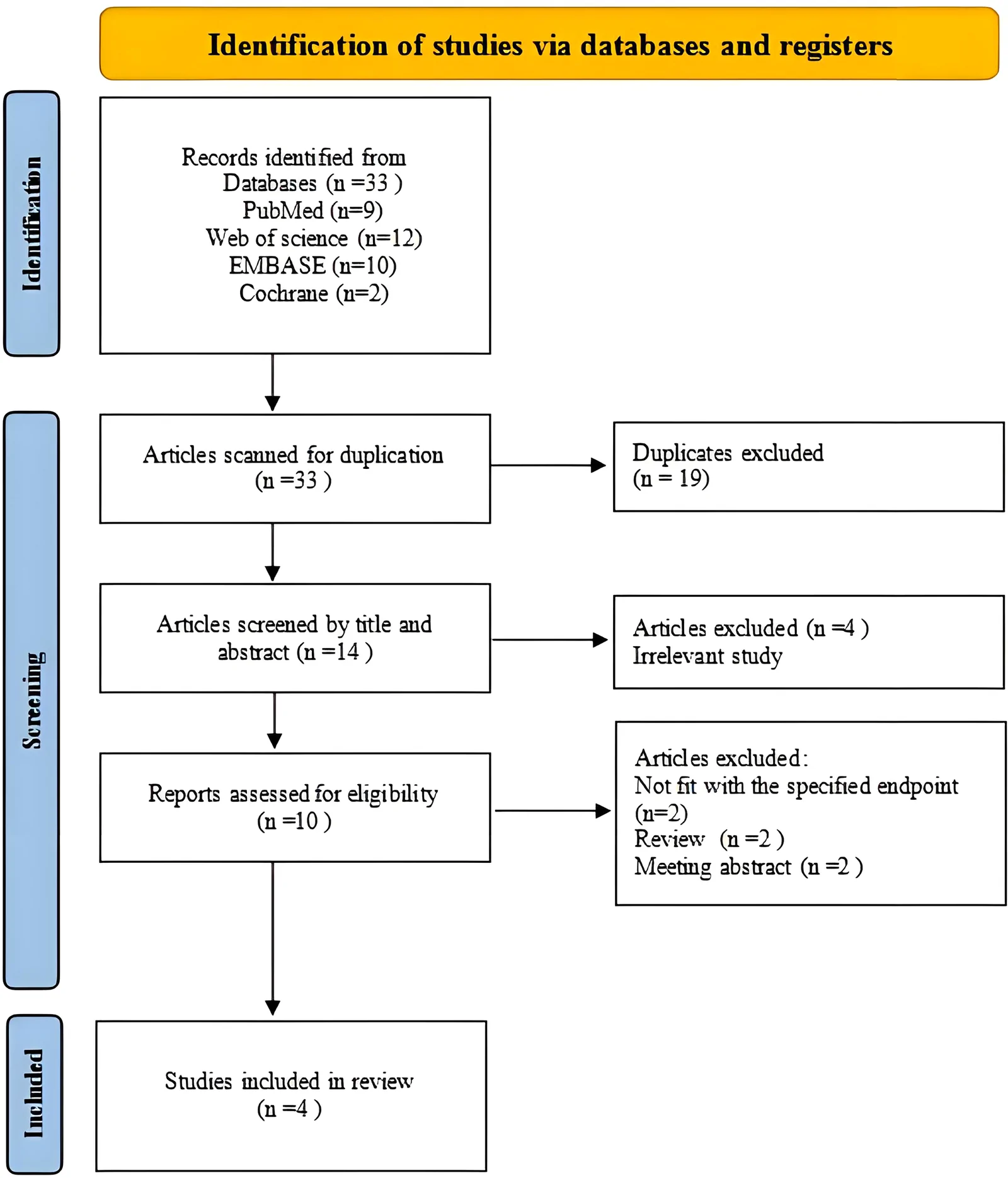
PRISMA flow diagram of the study selection process.

### 3.2 Baseline characteristics of included studies

The basic characteristics of all included studies were summarized and analyzed (**Table 1**). All enrolled studies were single-center cohort studies conducted in Chinese populations, covering a total of 1716 NPC participants. The age of enrolled patients ranged from 10 to 78 years, and all participants were newly diagnosed NPC cases without initial tumor invasion or distant metastasis at enrollment. All studies exhibited a consistent gender distribution bias, with male patients accounting for more than 65% of the total population. The earliest study cohort commenced patient enrollment in 2003, and two of the included studies focused primarily on patients with TNM stage III–IV NPC. The cut-off value of SUA in the population is determined based on the median. In terms of prognostic indicators, all four included studies reported complete data for OS, PFS, and DMFS. Additionally, three studies reported local recurrence-free survival (LRFS) outcomes; however, only descriptive statistical results were provided without available extractable HR values and 95% CI, which precluded LRFS from inclusion in the pooled quantitative analysis. The follow-up duration of the four cohorts ranged from 1.3 months to 71 months. Each eligible study reported complete baseline clinical information and valid survival prognostic data, which met the requirements for subsequent quantitative meta-analysis. The Newcastle-Ottawa Scale (NOS) was utilized to assess the methodological quality of the included studies, and all enrolled literatures were confirmed to be of medium-to-high quality, ensuring the reliability and validity of the pooled results. The detailed quality evaluation results of all included studies are shown in **Table 2**.

**Table 1:** Basic characteristics of the included literature.

Study	Country	Sample Size	Age (years)	Study design	Gender (M/F)	Study period	Study center	TNM stage	Treatment	Cutoff value of uric acid level (μmol/L)	Prevalence of hyperuricemia	Survival outcome	Follow-up (months)	Survival analysis	Pathologically diagnosed
Lin 2013 (22)	China	130	44 (18-76)	CS	88/42	2003-2005	Single	I-IVa	IMRT	301	NR	OS,PFS,DMFS	87 (6-101)	Multivariate	Without distant metastasis
Du 2016	China	1063	45 (14-78)	CS	813/250	2009-2012	Single	III-IV	IMRT	353.4	48.8%	OS,PFS,DMFS,LRFS	50 (1.3-70.7)	Multivariate	Non-disseminated
He 2026 (24)	China	182	50 (18-78)	CS	119/63	2017-2020	Single	III-IVb	IMRT,IMRT+CT	350	50%	OS,PFS,DMFS,LRFS	50 (30-71)	Multivariate	Without distant metastasis
Xu 2021 (25)	China	341	48 (10-78)	CS	231/110	2014-2017	Single	I-III	CT+CCRT	327	49%	OS,PFS,DMFS,LRFS	41 (3-62)	Multivariate	Locally advanced

CS, Cohort study; OS, Overall survival; PFS, Progression-free survival; DMFS, Distant metastasis-free survival; LRFS, locoregional relapse-free survival; IMRT, Intensity modulated radiation therapy; CCRT, Concurrent chemoradiotherapy; IC, Induction chemotherapy; NR, Not reported; M, Male; F, Female.

**Table 2:** Quality assessment of observational studies using the Newcastle-Ottawa scale (NOS).

Study	Study design	Selection	Comparability	Outcome/exposure	Overall score	Quality rating
Lin 2013 (22)	CS	3	2	3	8	High
Du 2016	CS	4	2	3	9	High
He 2026 (24)	CS	3	2	3	8	High
Xu 2021 (25)	CS	3	2	2	7	High

CS, Cohort study; According to the NOS scoring criteria (total score 0–9), a score of ≤ 3 is considered low quality, 4–6 as moderate quality, and ≥ 7 as high quality.

### 3.3 Association between SUA and overall survival

The results showed that SUA level was significantly associated with OS in NPC patients. The pooled HR was 0.245, and the 95%CI was 0.092–0.398. The 95%CI did not cross 1, indicating that the difference was statistically significant, which suggested that SUA level was an effective prognostic factor for the overall survival of NPC patients (**Figure 2a**). To further verify the stability of the prognostic correlation between SUA and OS, subgroup analyses were performed based on population age stratification, TNM stage, follow-up duration, and whether patients received intensity-modulated radiotherapy (IMRT). The subgroup analysis results consistently demonstrated that high SUA level may serve as a protective factor for OS in NPC patients across all stratified subgroups. Detailed data of subgroup analyses are presented in **Table 3**.

**Figure 2:**
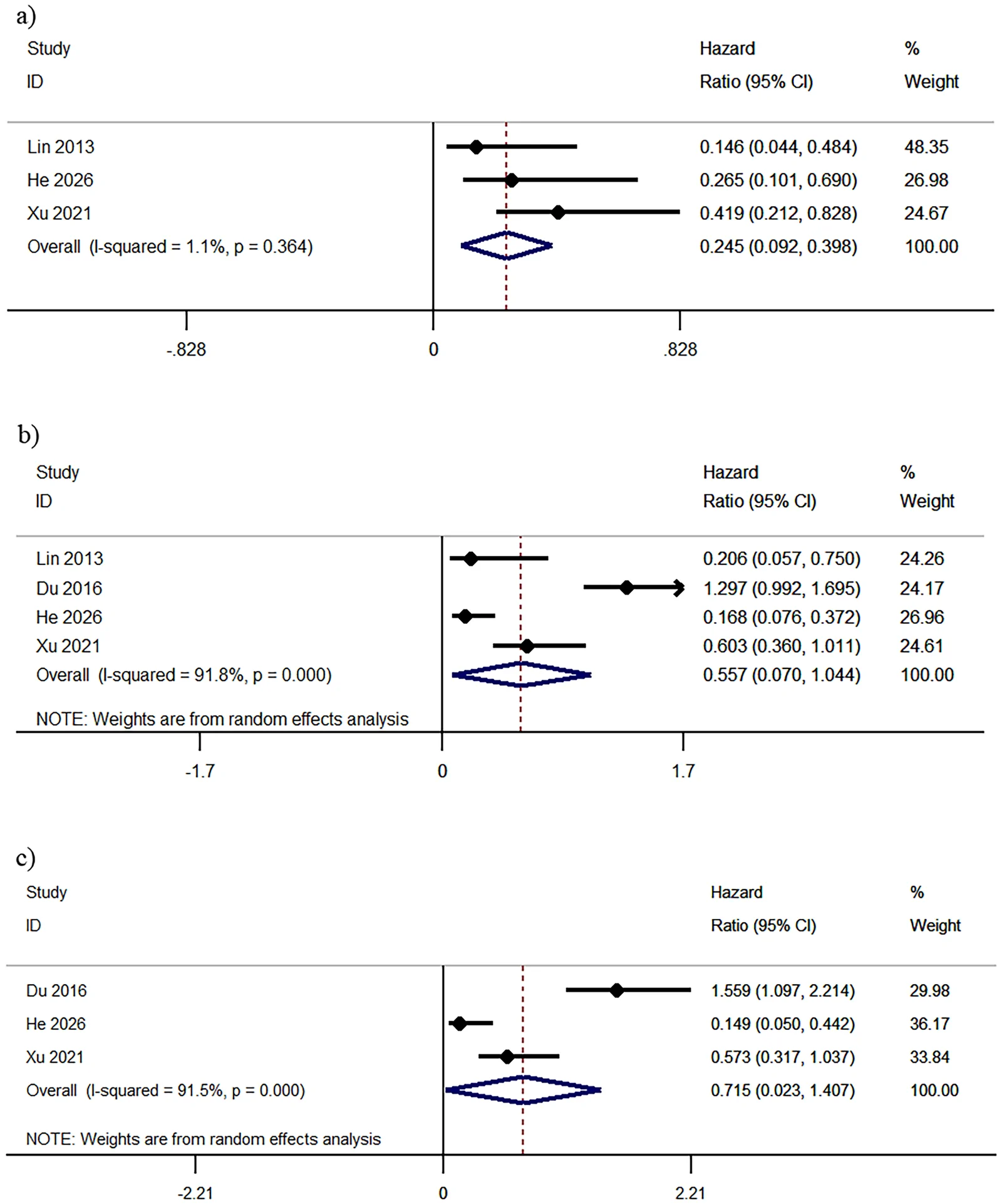
Forest plots for the prognostic role of SUC in patients with NPC. **(a)** OS; **(b)** PFS; **(c)** DMFS.

**Table 3:** Subgroup analysis of prognostic value of serum uric acid for OS in patients with NPC.

Subgroup	No. of studies	No. of patients	HR (95%CI)	Heterogeneity (I^2^, %)	P
Total	3	653	0.245 (0.092-0.398)	1.1	0.364
Median age (years)					
<45	1	130	0.146 (0.044-0.484)	–	–
≥45	2	523	0.339 (0.126-0.551)	0	0.479
TNM stage					
III-IV	1	182	0.265 (0.101-0.690)	–	–
Other	2	471	0.238 (0.059-0.417)	50	0.157
Median follow-up (months)					
<60	2	523	0.339 (0.126-0.551)	0	0.479
≥60	1	130	0.146 (0.044-0.484)	–	–
IMRT treatment					
Yes	2	312	0.189 (0.012-0.365)	0	0.526
No	1	341	0.419 (0.212-0.828)	–	–

### 3.4 Association between SUA and PFS

The pooled HR of SUA level and PFS in NPC patients was 0.557, with a 95%CI of 0.070–1.044. The 95%CI crossed the invalid value of 1, indicating no significant statistical correlation between SUA level and PFS of NPC patients. Heterogeneity test revealed a high heterogeneity across included studies, with an I² value of 91.8%. Given the significant high heterogeneity, a random-effects model was adopted for the pooled analysis (**Figure 2b**). To explore the potential sources of heterogeneity, subgroup analyses were further conducted based on sample size, age stratification, male proportion, TNM stage, and tumor invasion status. Subgroup results demonstrated that the non-significant association between SUA and PFS was consistent with the overall pooled results in subgroups of large sample size, advanced age group, high male proportion, TNM stage III–IV, and non-invasive NPC patients, with no significant correlation identified between SUA levels and PFS. Notably, subgroup analyses indicated that high SUA level might serve as a protective factor for PFS in patients with younger age, early TNM stage, and long follow-up duration. The high post-treatment serum uric acid level might be associated with a favorable PFS. Detailed subgroup analysis data for PFS are presented in **Table 4**.

**Table 4:** Subgroup analysis of prognostic value of serum uric acid for PFS in patients with NPC.

Subgroup	No. of studies	No. of patients	HR (95%CI)	Heterogeneity (I^2^, %)	P
Total	4	1716	0.557 (0.070-1.044)	91.8	<0.001
Sample size					
<1000	3	653	0.303 (0.041-0.565)	65	0.057
≥1000	1	1063	1.297 (0.992-1.695)	–	–
Median age (years)					
<45	1	130	0.206 (0.057-0.750)	–	–
≥45	3	1586	0.675 (0.017-1.334)	94.4	<0.001
Male proportion					
<75%	3	653	0.303 (0.041-0.565)	65	0.057
≥75%	1	1063	1.297 (0.992-1.695)	–	–
TNM stage					
III-IV	2	1245	0.721 (0.385-1.827)	97	0.102
Other	2	471	0.409 (0.020-0.798)	62.7	<0.001
Median follow-up (months)					
<60	3	1586	0.675 (0.017-1.334)	94.4	<0.001
≥60	1	130	0.206 (0.057-0.750)	–	–
IMRT treatment					
Yes	3	1375	0.546 (0.114-1.207)	94.1	<0.001
No	1	341	0.603 (0.360-1.011)	–	–
Pathologically diagnosed					
Non-disseminated	1	1063	1.297 (0.992-1.695)	–	–
Other	3	653	0.303 (0.041-0.565)	65	0.057
SUC classification					
Pretreatment	1	1063	1.297 (0.992-1.695)	–	–
Post-treatment	3	653	0.303 (0.041-0.565)	65	0.057

### 3.5 Association between SUA and DMFS

The pooled HR of SUA level and DMFS was 0.715, and the 95%CI was 0.023–1.407. Similarly, the 95%CI spanned 1, demonstrating that there was no significant association between SUA level and DMFS of NPC patients. Heterogeneity test indicated substantial heterogeneity among included studies, with an I² value of 91.5%. Accordingly, a random-effects model was applied for the pooled DMFS analysis (**Figure 2c**). To explore the potential source of heterogeneity and validate the robustness of the primary outcome, subgroup analysis stratified by TNM stage was further performed. The results showed that TNM stage exerted no substantial influence on the non-significant association between SUA level and DMFS, and the overall analytical conclusion remained consistent across different TNM subgroups. The high post-treatment serum uric acid level might be associated with a favorable DMFS. Detailed subgroup analysis data for DMFS are presented in **Table 5**.

**Table 5:** Subgroup analysis of prognostic value of serum uric acid for DMFS in patients with NPC.

Subgroup	No. of studies	No. of patients	HR (95%CI)	Heterogeneity (I^2^, %)	P
Total	3	1586	0.715 (0.023-1.407)	91.5	<0.001
Sample size					
<1000	2	523	0.333 (0.079-0.745)	75.7	0.043
≥1000	1	1063	1.559 (1.097-2.214)	–	–
Male proportion					
<75%	2	523	0.333 (0.079-0.745)	75.7	0.043
≥75%	1	1063	1.559 (1.097-2.214)	–	–
TNM stage					
III-IV	2	1245	0.829 (0.552-2.210)	95.4	<0.001
Other	1	341	0.573 (0.317-1.037)	–	–
IMRT treatment					
Yes	2	1245	0.829 (0.552-2.210)	95.4	<0.001
No	1	341	0.573 (0.317-1.037)	–	–
Pathologically diagnosed					
Non-disseminated	1	1063	1.559 (1.097-2.214)	–	–
Other	2	523	0.333 (0.079-0.745)	75.7	0.043
SUC classification					
Pretreatment	1	1063	1.559 (1.097-2.214)	–	–
Post-treatment	2	523	0.333 (0.079-0.745)	75.7	0.043

### 3.6 Sensitivity analysis

Sequential leave-one-out sensitivity analysis was performed to verify the robustness of the pooled prognostic results for OS, PFS, and DMFS. After iterative exclusion of each individual study separately, the recalculated pooled HR values and 95% CI for all survival endpoints did not undergo prominent fluctuations or directional changes. Consistent with our overall findings, no single enrolled study dominated or altered the statistical outcomes of the present meta-analysis, further confirming that the synthetic results regarding the prognostic association between SUA and NPC survival were highly stable and robust (**Figure 3**).

**Figure 3:**
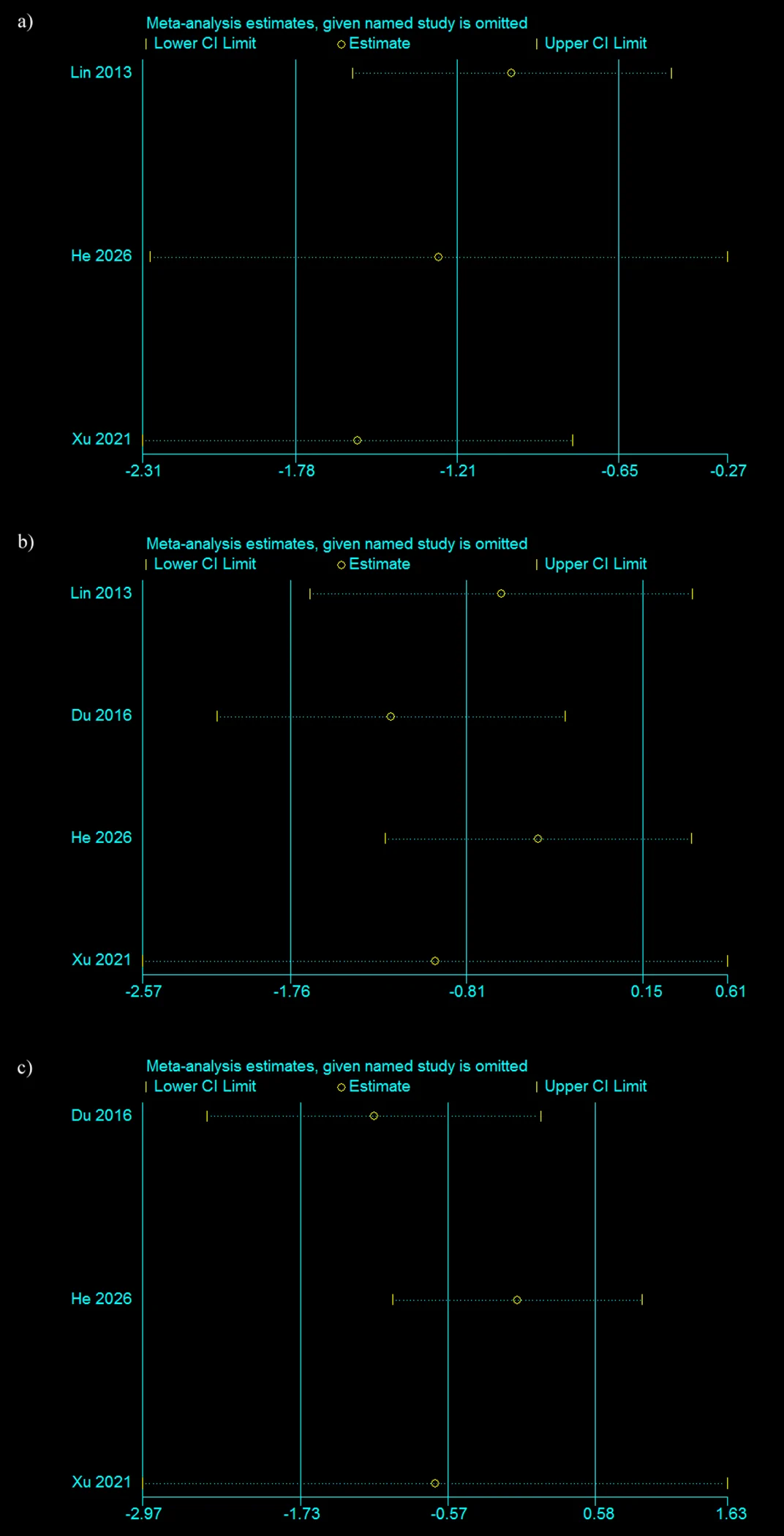
Sensitivity analysis. **(a)** OS; **(b)** PFS; **(c)** DMFS.

## 4 Discussion

This study is the first comprehensive and systematic meta-analysis to quantitatively elucidate the prognostic significance of SUA levels in NPC patients. This meta-analysis incorporated four qualified single-center cohort studies encompassing 1716 NPC participants, overcoming the limitations of small sample sizes, inconsistent findings, and scattered evidence from individual original studies. By integrating overall pooled estimation, stratified subgroup analysis, sensitivity verification, and publication bias assessment, our study yielded evidence regarding the differential associations between SUA and multiple survival endpoints of NPC, providing evidence-based insights for NPC prognostic evaluation and clinical risk stratification. The core finding of the current meta-analysis is that elevated SUA serves as a significant protective biomarker for long-term overall survival in NPC patients. The pooled results demonstrated that higher SUA was independently correlated with favorable OS outcomes with a stable pooled HR of 0.245 (95% CI: 0.092–0.398), and subsequent subgroup analyses further validated the stability of this protective effect across diverse stratifications, including different age groups, TNM stages, follow-up durations, and IMRT treatment status.

Consistent with emerging evidence in multiple malignancies, the protective prognostic role of SUA in NPC can be biologically explained by its dual regulatory function in oxidative stress and tumor immune microenvironment. Accumulating evidence has demonstrated that serum xanthine oxidoreductase (XOR) is a pivotal producer of reactive oxygen species (ROS). The pioneering work by Ames et al. first established that serum SUA is a highly efficient scavenger of singlet oxygen and hydroxyl radicals, laying the theoretical foundation for its identification as a potential protective factor against human cancer (26). A key mechanism for the bodily protection of SUA is its antioxidant activity, which is estimated to account for 50% of human blood’s total antioxidant capacity (27). SUA functions as a powerful antioxidant by scavenging reactive oxygen species (ROS), preventing peroxynitrite-induced protein nitrosylation and lipid/protein peroxidation, and inactivating tetrahydrobiopterin (28). The burst of ROS derived from XOR disturbs cellular redox homeostasis, and further participates in the regulatory networks of diverse biological processes such as inflammatory activation, cell proliferation and neoplastic transformation (29, 30). As a critical endogenous antioxidant, physiological and moderately elevated SUA can scavenge reactive oxygen species (ROS), reduce oxidative DNA damage, and alleviate persistent inflammatory infiltration in tumor tissues, thereby inhibiting malignant proliferation and distant progression of NPC cells. Building on prior knowledge of SLC2A9 as a typical SUA transporter, previous study further demonstrated that this molecule serves as a key downstream target of p53. The study also clarified that this p53-SLC2A9 axis plays a protective role in mitigating ROS-induced damage and restraining human cancer pathogenesis (31). Furthermore, interventional experiments that administered exogenous SUA to healthy human volunteers have demonstrated that SUA can effectively reduce the production of reactive oxygen species (ROS) (32). Previous investigations have additionally demonstrated that SUA confers protective effects against the progression of selected solid tumors (such as hepatocellular carcinoma) through orchestrating glutathione metabolic reprogramming, mitigating lipid peroxidation and blocking ferroptosis, an iron-driven programmed cell death pathway (15). This inherent mechanistic duality emphasizes the essential value of comprehensively dissecting the relationship between SUA and oncogenesis risk. Moreover, higher SUA levels often reflect better baseline metabolic function and systemic immune status in NPC patients, which may partially account for superior long-term survival compared with patients with decreased SUA levels.

Notably, our stratified analyses revealed distinct associations of SUA with different survival endpoints of NPC, indicating a stage-specific prognostic value of SUA in NPC progression. No significant overall correlation was observed between SUA levels and PFS or DMFS in the overall population. Consistent with prior research conclusions, this review failed to identify convincing, high-grade evidence that supports a definitive causal effect of SUA levels on cancer-related outcomes (5). Of note, high statistical heterogeneity was detected in both PFS (I² = 91.8%) and DMFS (I² = 91.5%) analyses, suggesting prominent clinical and methodological inconsistencies across enrolled studies. Further subgroup analyses successfully identified potential sources of heterogeneity and revealed meaningful differential results. For PFS, the non-significant association remained stable in subgroups of large sample size, advanced age, high male proportion, stage III–IV disease, and non-invasive NPC. However, elevated SUA exhibited a potential protective trend for PFS in patients with younger age, early TNM stage, and prolonged follow-up duration. For DMFS, TNM stratification did not alter the overall null association between SUA and distant metastasis, further confirming the reliability of the primary DMFS result. The inconsistent prognostic performance of SUA across different survival endpoints may be attributable to the complex and multifactorial nature of NPC recurrence and metastasis (33). Tumor local progression and distant metastasis are predominantly driven by TNM stage, radiotherapy response, tumor pathological differentiation, and individual antitumor immunity, whereas peripheral metabolic indicators such as SUA exert limited independent predictive effects on short-term disease progression (24).

From a clinical perspective, SUA possesses unique advantages as a promising auxiliary prognostic biomarker for NPC. Compared with traditional prognostic indicators relying on imaging examination and pathological staging, SUA detection is non-invasive, inexpensive, readily available, and routinely tested in clinical practice, enabling dynamic and convenient baseline risk assessment for NPC patients before treatment. Based on our findings, post-treatment SUA level can serve as a complementary metabolic biomarker to optimize the existing NPC prognostic evaluation system. Clinically, NPC patients with reduced baseline SUA should be recognized as high-risk populations for poor long-term overall survival, warranting intensified individualized treatment regimens, closer surveillance, and prolonged follow-up strategies to improve long-term survival benefits. Meanwhile, considering the subgroup-specific predictive characteristics of SUA for PFS, targeted attention should be paid to young patients with early-stage NPC during clinical follow-up (34).

Several limitations of the present study should be acknowledged. First, although we conducted comprehensive subgroup analyses to explore heterogeneity sources, the limited number of eligible studies failed to further explore the prognostic value of SUA in more refined stratifications, such as different radiotherapy modalities, chemotherapy regimens, and precise age stratifications. Second, since the included studies lack the LRFS follow-up outcomes, it is impossible to evaluate the association between SUA and local recurrence of NPC. Third, the evidence included in this study lacks data on the dynamic changes of SUA during antitumor therapy, and its prognostic significance remains unclear. Fourth, all included cohorts were derived from Chinese populations, which may limit the generalizability of our conclusions to non-endemic regions and other ethnic populations. Finally, due to the inclusion of only 4 literatures in this meta-analysis, Egger publication bias test was not performed in this study. Sensitivity analysis confirmed the robustness of pooled estimates, and small-sample bias would not fundamentally alter the core conclusion. Further bias assessment is warranted when more relevant studies become available.

In summary, the current meta-analysis comprehensively clarified the differential prognostic roles of SUA in NPC survival. The findings indicate that SUA is a reliable protective factor for overall survival in NPC patients, while its predictive value for tumor progression and distant metastasis is limited and population-dependent. Further large-sample, multi-center, prospective longitudinal studies are urgently required to validate our conclusions, explore the underlying molecular mechanisms of SUA regulating NPC progression, and promote the clinical application of SUA in individualized prognostic management of NPC.

## 5 Conclusions

In conclusion, this first systematic meta-analysis clarifies that SUA level is a significant independent prognostic factor for overall survival in NPC patients, and high SUA level is closely related to favorable long-term overall survival. However, there is no sufficient evidence to prove that SUA level is associated with PFS and DMFS of NPC patients. As a simple, economical and non-invasive peripheral blood biomarker, SUA may provide a reference for prognostic risk stratification and individualized management of NPC. Considering the limitations of existing studies, further large-sample, multi-center, high-quality prospective cohort studies are required to verify the current conclusions and explore the potential clinical application value of SUA in NPC prognosis prediction.

## Data Availability

The original contributions presented in the study are included in the article/**Supplementary Material**. Further inquiries can be directed to the corresponding authors.
